# *Posidonia oceanica* (L.) Delile Is a Promising Marine Source Able to Alleviate Imiquimod-Induced Psoriatic Skin Inflammation

**DOI:** 10.3390/md22070300

**Published:** 2024-06-28

**Authors:** Laura Micheli, Marzia Vasarri, Donatella Degl’Innocenti, Lorenzo Di Cesare Mannelli, Carla Ghelardini, Antiga Emiliano, Alice Verdelli, Marzia Caproni, Emanuela Barletta

**Affiliations:** 1 Department of Neuroscience, Psychology, Drug Research and Child Health (NEUROFARBA), Pharmacology and Toxicology Section, University of Florence, 50139 Firenze, Italy; laura.micheli@unifi.it (L.M.); lorenzo.mannelli@unifi.it (L.D.C.M.); carla.ghelardini@unifi.it (C.G.); 2 Department of Experimental and Clinical Biomedical Sciences “Mario Serio”, Biochemistry Section, University of Florence, 50134 Firenze, Italy; marzia.vasarri@unifi.it (M.V.); donatella.deglinnocenti@unifi.it (D.D.); 3 CIBM, Applied Ecology and Marine Biology Interuniversity Centre “G. Bacci”, 57128 Livorno, Italy; 4 Department of Health Sciences, Dermatology Section, University of Florence, 50125 Firenze, Italy; emiliano.antiga@unifi.it (A.E.); marzia.caproni@unifi.it (M.C.); 5 Central Tuscany Local Health Authority, Department of Multidimensional Medicine, Immuno-Rheumatology and Infectious Diseases Area, Dermatology SOC, Dermatological Rare Diseases SOS, 50125 Firenze, Italy; alice.verdelli@hotmail.it; 6 Department of Experimental and Clinical Biomedical Sciences “Mario Serio”, Experimental Pathology and Oncology Section, University of Florence, 50134 Firenze, Italy

**Keywords:** *Posidonia oceanica*, Imiquimod, inflammation, psoriasis, IL-17, IL-23, TNF-α, Lipocalin-2

## Abstract

Psoriasis is a chronic immune-mediated inflammatory cutaneous disease characterized by elevated levels of inflammatory cytokines and adipokine Lipocalin-2 (LCN-2). Recently, natural plant-based products have been studied as new antipsoriatic compounds. We investigate the ability of a leaf extract of the marine plant *Posidonia oceanica* (POE) to inhibit psoriatic dermatitis in C57BL/6 mice treated with Imiquimod (IMQ). One group of mice was topically treated with IMQ (IMQ mice) for 5 days, and a second group received POE orally before each topical IMQ treatment (IMQ-POE mice). Psoriasis Area Severity Index (PASI) score, thickness, and temperature of the skin area treated with IMQ were measured in both groups. Upon sacrifice, the organs were weighed, and skin biopsies and blood samples were collected. Plasma and lesional skin protein expression of IL-17, IL-23, IFN-γ, IL-2, and TNF-α and plasma LCN-2 concentration were evaluated by ELISA. PASI score, thickness, and temperature of lesional skin were reduced in IMQ-POE mice, as were histological features of psoriatic dermatitis and expression of inflammatory cytokines and LCN-2 levels. This preliminary study aims to propose *P. oceanica* as a promising naturopathic anti-inflammatory treatment that could be introduced in Complementary Medicine for psoriasis.

## 1. Introduction

Psoriasis is an immune-mediated chronic inflammatory disease of the skin with an estimated worldwide prevalence of about 2–3% which results from interactions between genetic predisposition and environmental stimuli. Besides the dermatological manifestations, it can also affect other organs. Therefore, psoriasis can be considered a systemic inflammatory disease rather than merely a cutaneous disease. Psoriasis severity is assessed and graded by the Psoriasis Area and Severity Index (PASI) [1], which evaluates the percentage of the affected body area and measures the intensity of redness (erythema), infiltration (thickness), and desquamation (scaliness). Histologically, skin lesions are characterized by an inflammatory infiltration of the dermis and epidermis consisting of macrophages, lymphocytes, neutrophils, and dendritic cells, and by an increase in keratinocyte proliferation, acanthosis (thickening of the stratum spinosum), and parakeratosis (cell nuclei within stratum corneum) [2]. 

A pivotal role in the pathogenesis of the psoriatic disease is played by cytokines such as tumor necrosis factor alpha (TNF-α), interleukin-23 (IL-23, composed of the IL-12B p40 subunits shared with IL-12, and the IL-23A p19 subunit), and the interleukin-17 family (IL-17A through IL-17F), secreted by activated keratinocytes, by dendritic Langerhans cells, and by Th17 and Th22 subpopulations of T helper lymphocytes [3,4,5]. Moreover, IL-17 can act with TNF-α to induce amplification of the initial inflammatory trigger by activating the NF-κB pathway [6,7]. Furthermore, TNF-α and the IL-23/IL-17 axis are among the main targets of biological therapies that use monoclonal antibodies against these cytokines [8,9]. In the last few years, several studies have also revealed that adipokine Lipocalin-2 (LCN-2) is highly expressed in psoriatic patients and in psoriasis mouse models [10]. LCN-2, also known as neutrophil gelatinase-associated lipocalin (NGAL), is a 25 kDa protein covalently bound to the matrix metalloproteinase 9. LCN-2 participates in several biological functions, such as immune response, cell growth, iron transport, and synthesis of inflammatory mediators. LCN-2 plays many roles in the pathogenesis of psoriasis both in the differentiation and proliferation of keratinocytes and in the recruitment of inflammatory cells such as lymphocytes and neutrophils via the IL-23/IL-17 axis [11]; thus, LCN-2 might be considered a marker of psoriasis [12]. 

In recent years, a mouse psoriasis-like model has been developed by topical application to the skin surface of the imidazoquinoline compound Imiquimod (IMQ) [13,14,15], a well-known ligand of Toll-like receptors 7 and 8 [16]. The IMQ-induced mouse model of psoriasis displays skin lesions closely related to human cutaneous psoriasis, clinically characterized by erythema, skin thickening, and scaling. Typical histological alterations such as acanthosis and parakeratosis, and inflammatory infiltrates of lymphocytes, macrophages, neutrophils, and dendritic cells are also reproduced. In addition, in this model, IMQ-treated skin displays a higher expression of IL-17 and IL-23 than untreated skin, indicating the involvement of the IL-23/IL-17 axis. Therefore, the IMQ-induced mouse model of psoriasis is widely used to study the pathogenesis of psoriatic skin lesions as well as the development of new therapies [13,15]. The therapeutical management of psoriasis provides different approaches depending on disease severity. Mild psoriasis is treated with topical therapies and phototherapy, while moderate and severe psoriasis are treated with systemic therapy, including biological agents against specific cytokines. [17,18]. Although biologics are usually considered safer than conventional immunosuppressive treatments, not all psoriatic patients are good candidates for biological therapies. For example, patients with malignant neoplasms and with active infections or systemic diseases cannot be treated with biologics since biological agents can induce side effects such as infections and malignancies [19,20,21]. 

In recent years, several natural products from plants have been considered as new potential anti-psoriatic agents with the aim of finding a complementary/alternative drug characterized by the absence of long-term toxicity, leading to better patient compliance than conventional therapies [22,23]. 

Although plants have historically been one of the main sources of pharmacological substances, in recent years, more attention has been paid to the marine world as an exceptional reservoir of new bioactive molecules that are safe and effective for human health, including anti-inflammatory agents [24]. 

*Posidonia oceanica* L. Delile is a Mediterranean angiosperm traditionally used as a medicinal plant for the treatment of various human diseases [25]. The healing properties of *P. oceanica* have been described in ancient Egypt, where it was said to be used for sore throats and skin problems [26]. Recently, phytotherapy has focused attention on *P. oceanica* for its bioactive properties and, in addition to its ecological importance, as a potential source of new drugs [25]. New findings on the bioactive properties of *P. oceanica* make this plant a promising source of natural therapeutic products for human health [25]. Studies conducted on in vitro cell models and in vivo animal models pave the way for research into new alternative and complementary therapeutic strategies based on *P. oceanica* against a wide range of pathological conditions.

Our laboratory has revealed that the hydroalcoholic extract obtained from *P. oceanica* leaves (POE) has no toxicity both in vitro and in vivo and, among other activities, has anti-inflammatory properties [27,28]. Indeed, in the murine macrophage RAW264.7 cell line stimulated with lipopolysaccharide, POE has inhibited intracellular ROS levels and the activity of the proinflammatory enzymes iNOS and COX-2, as well as the NF-κB signalling pathway [27]. Furthermore, in CD1 mice, oral administration of POE has inhibited carrageenan-induced inflammation by reducing pain and paw edema, by suppressing tissue myeloperoxidase activity and reducing the tissue concentration of the proinflammatory cytokines IL-1β and TNF-α [28], suggesting that oral administration of POE may exert anti-inflammatory effects both locally and systemically.

Given the demonstrated anti-inflammatory properties of POE, this preliminary study aims to verify the ability of POE to prevent the main inflammatory aspects of psoriatic-like lesions in the IMQ-induced mouse model, thus providing a new potential source of natural compounds to be used in alternative medicine of psoriasis.

## 2. Results

### 2.1. Body and Internal Organ Weight

**Figure 1:**
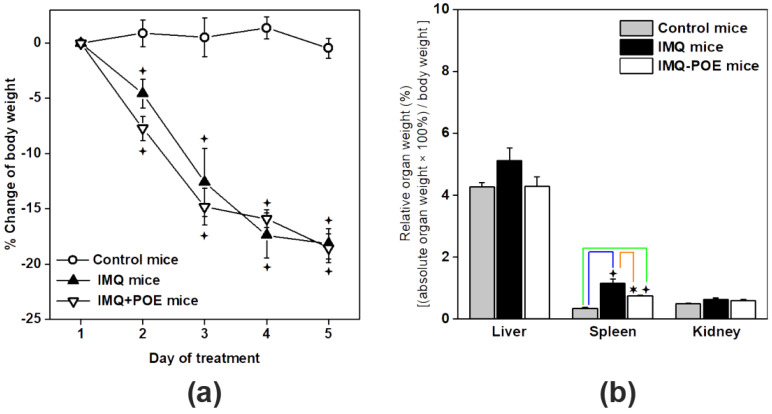
Body weight and organ weight changes. (**a**) Changes in body weight are expressed as a percentage of the initial value (day 1) in control mice (white circle), IMQ mice (black triangle), and IMQ-POE mice (white triangle). Values are mean ± SEM (*n* = 6/group) measured at different time points. (**b**) Changes in liver, spleen, and kidney weights are expressed as organ-relative weight at the end of the experimental period (day 5) in control mice (grey bar), IMQ mice (black bar), and IMQ-POE mice (white bar). The bar graph represents the mean ± SEM (*n* = 6/group). ✦ *p* < 0.05 IMQ mice or IMQ-POE mice vs. control mice by one-way ANOVA with Bonferroni post hoc test; ✶ *p* < 0.05 IMQ-POE mice vs. IMQ mice by one-way ANOVA with Bonferroni post hoc test; if it is not indicated, the differences are statistically non-significant.

To verify the systemic effects of topical IMQ treatment, the body weight in control mice and in experimental mice was recorded daily during the 5-day period. As shown in 1a, topical application of IMQ induced a body weight loss of less than 18% of the initial body weight in both IMQ and IMQ-POE mice; body weight in control mice was unaffected by topical application of vehicle cream and oral administration with POE vehicle during all five days. In addition, at the end of the experimental period (day 5), the organ’s relative weight was evaluated. As shown in Figure 1b, the relative weight of the liver and kidney in both IMQ and IMQ-POE mice did not differ from the relative weight of the liver and kidney of control mice. However, during the topical application of IMQ, both groups of experimental mice displayed a statistically significant increase (*p* < 0.05) in the relative weight of the spleen compared to the control group (IMQ mice, 1.1 ± 0.1; IMQ-POE mice 0.7 ± 0.02; control mice 0.3 ± 0.02). However, IMQ-POE mice displayed a lesser increase in spleen weight than IMQ mice (0.7 ± 0.02 versus 1.1 ± 0.1, *p* < 0.05). These results suggest that topical IMQ treatment with or without oral administration of POE has no toxic effect since there was no change in the organ weight-to-body weight ratio of the liver and kidney compared to the control group. In addition, although topical application of IMQ induces splenomegaly according to previous findings [14,29,30,31], a recent study has revealed that the spleen is not directly involved in the development of psoriatic lesions in IMQ-treated mice [31].

### 2.2. Clinical Evaluation of Skin Inflammation

**Figure 2:**
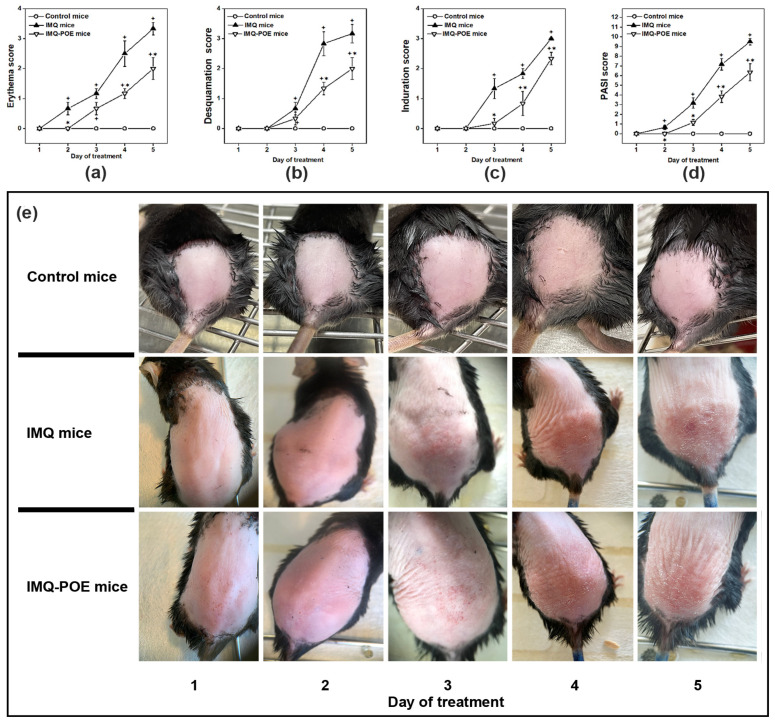
Evaluation of psoriatic skin inflammation. (**a**) Erythema score, range 0–4. (**b**) Desquamation score, range 0–4. (**c**) Induration score, range 0–4. (**d**) PASI cumulative index (sum of the three a, b, and c scores), range from 0 to 12. The line graph represents the mean of each score ± SEM (*n* = 6/group) measured at different time points in control mice (white circle), IMQ mice (black triangle), and IMQ-POE mice (white triangle). ✦ *p* < 0.05 IMQ mice or IMQ-POE mice vs. control mice by one-way ANOVA with Bonferroni post hoc test; ✶ *p* < 0.05 IMQ-POE mice vs. IMQ mice by one-way ANOVA with Bonferroni post hoc test; if it is not indicated, the differences are statistically non-significant. (**e**) Representative pictures of the dorsal treated skin area of control mice, IMQ mice, and IMQ-POE mice during each day of the treatment period.

To clinically assess the severity of skin inflammation during topical application of IMQ and to verify whether oral administration of POE could improve inflammatory status, PASI score, thickness, and temperature of the treated skin area were evaluated. PASI score was equal to 0 in control mice during all five days (Figure 2d). When compared to control mice, after 24 h from the first application of IMQ (day 2), erythema had already appeared in IMQ mice (Figure 2a), while desquamation and induration were evident after 48 h (day 3, Figure 2b,c) with a PASI score > 3 (PASI = 3.1 ± 0.5, day 3, Figure 2d). At the end of the experimental period, the PASI score was 9.5 in IMQ mice (PASI = 9.5 ± 0.3, day 5, Figure 2d). During oral administration of POE, signs of skin inflammation related to IMQ topical application were less severe; erythema appeared after 48 h in comparison to control mice (day 3, Figure 2a), with a PASI score lower than the PASI score observed in IMQ mice (PASI = 1.1 ± 0.3 versus PASI = 3.1 ± 0.5, *p* < 0.05, day 3, Figure 2d), while desquamation and induration appeared after 72 h in comparison to control mice (day 4, Figure 2b,c), with a PASI score still lower than the PASI score observed in IMQ mice (PASI = 3.8 ± 0.6 versus PASI = 7.1 ± 0.6, *p* < 0.05, day 4, Figure 2d). At the end of the experimental period, the PASI score in IMQ-POE mice was still lower than the PASI observed in IMQ mice (PASI = 6.3 ± 0.8 versus PASI = 9.5 ± 0.3, *p* < 0.05, day 5, Figure 2d). Figure 2e shows clinical pictures of psoriatic lesions developed during each day in IMQ mice and the attenuation of lesional severity during oral administration of POE. Application of vehicle cream in control mice never induces any skin alteration.

**Figure 3:**
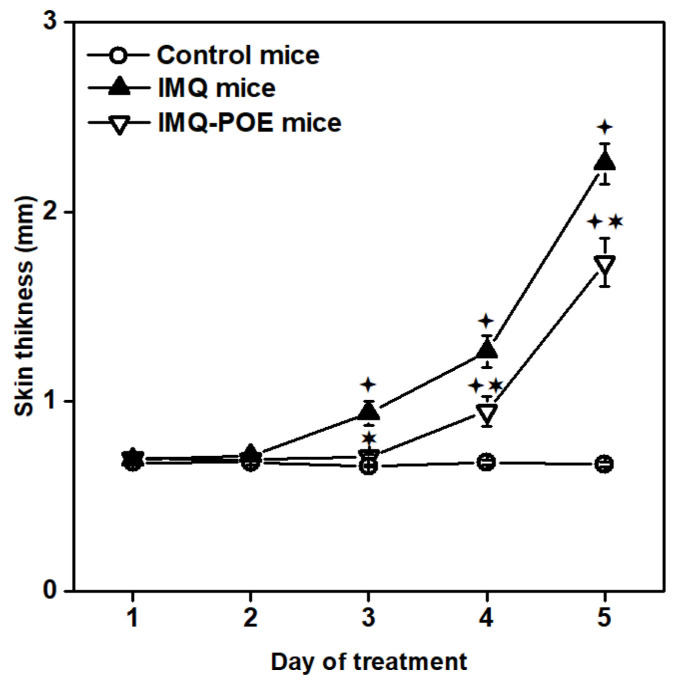
Measurement of dorsal skin thickness. Double-fold dorsal skin thickness was measured in the dorsal treated area by an engineer’s micrometer in control mice (white circle), IMQ mice (black triangle), or IMQ-POE mice (white triangle). Values are reported in millimeters and expressed as mean ± SEM (*n* = 6/group). ✦ *p* < 0.05 IMQ mice or IMQ-POE mice vs. control mice by one-way ANOVA with Bonferroni post hoc test; ✶ *p* < 0.05 IMQ-POE mice vs. IMQ mice by one-way ANOVA with Bonferroni post hoc test; if iAt is not indicated, the differences are statistically non-significant.

Since the thickness of the inflamed skin area is related to the intensity of oedema, changes in the double-fold thickness of the treated skin area were recorded during the 5 days of treatment. In control mice, the thickness of skin treated with vehicle cream did not change over the 5 days, while the thickness of Imiquimod-treated skin was increased in both IMQ and IMQ-POE mice (Figure 3). However, in IMQ mice, the thickness of the treated skin area was higher than in IMQ-POE mice after 48 h from topical application of IMQ (Figure 3, day 3, 0.9 ± 0.06 mm versus 0.7 ± 0.03 mm, *p* < 0.05) and it remained at higher levels until the end of treatment. These results are consistent with a lower intensity of skin inflammation during POE treatment. 

**Figure 4:**
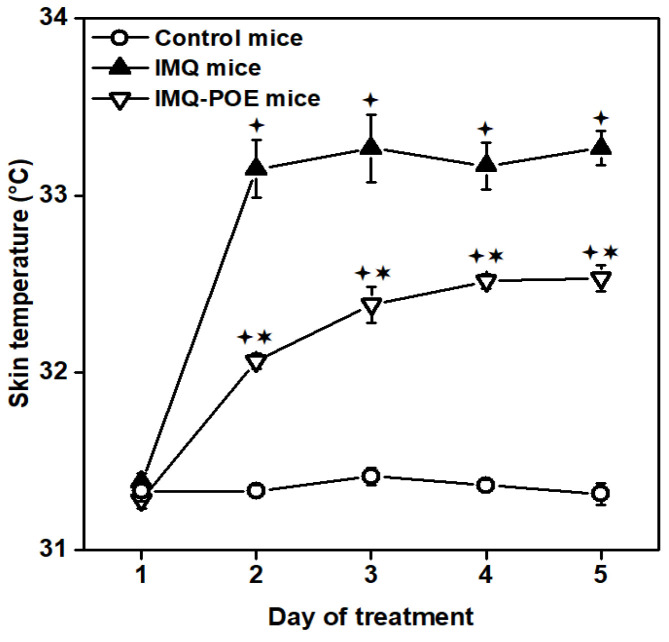
Changes in dorsal skin temperature. Skin temperature was measured in the dorsal treated area of control mice (white circle), IMQ mice (black triangle), or IMQ-POE mice (white triangle) by an infrared camera during the 5 days of treatment. Values are reported in °C and expressed as mean ± SEM (*n* = 6/group). ✦ *p* < 0.05 IMQ mice or IMQ-POE mice vs. control mice by one-way ANOVA with Bonferroni post hoc test; ✶ *p* < 0.05 IMQ-POE mice vs. IMQ mice by one-way ANOVA with Bonferroni post hoc test; if it is not indicated, the differences are statistically non-significant.

Finally, the temperature of the treated skin area was measured, since temperature increase is one of the four cardinal signs of inflammation. While skin temperature in control mice did not change over the 5 days, after 24 h from IMQ topical application (day 2), both groups of IMQ and IMQ-POE mice displayed an increase in skin temperature; however, in the treated skin area, the temperature in IMQ mice was higher than in IMQ-POE mice (Figure 4, 33.1 ± 0.3 °C versus 32.0 ± 0.04 °C, *p* < 0.05) and it remained higher in IMQ mice than in IMQ-POE mice for the subsequent days. These results confirm that POE oral administration improves inflammatory status induced by topical application of IMQ.

### 2.3. Histological Examination and Immunodetection of Macrophages and Lymphocytes

**Figure 5:**
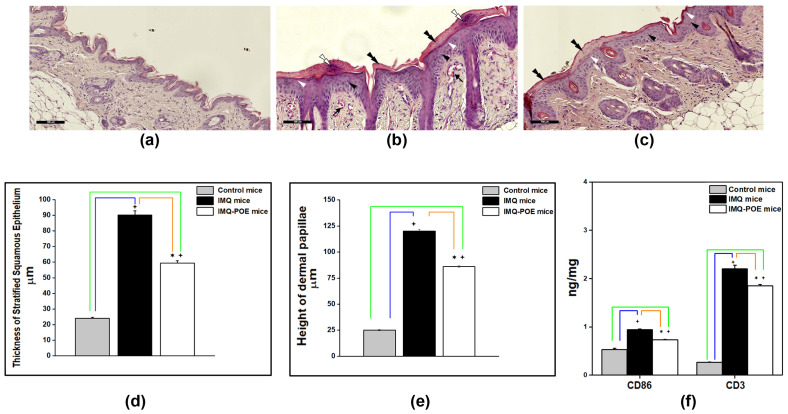
Histological examination. Representative pictures of hematoxylin and eosin-stained skin sections obtained from the dorsal skin area treated with (**a**) vehicle cream (control mice); (**b**) Imiquimod (IMQ mice); (**c**) Imiquimod and oral administration of POE (IMQ-POE mice). Scale bar, 100 µm. Black arrowheads indicate epidermal hyperplasia; double black arrowheads indicate hyperkeratosis and parakeratosis; white arrowheads indicate agranulosis; black arrows indicate dilatation and congestion of the dermal capillaries; double white arrowheads indicate Munro’s micro abscesses. Microscopic evaluation of the thickness of stratified squamous epithelium (**d**) and the height of dermal papillae (**e**), in skin sections of control mice (grey bar), skin sections of IMQ mice (black bar), and skin sections of IMQ-POE mice (white bar); values are reported in micrometres and expressed as mean ± SEM. (**f**) The levels of CD86 marker of inflammatory macrophages and CD3 marker of T lymphocytes were detected by ELISA in the treated dorsal skin area; values are reported in ng/mg of tissue and expressed as mean ± SEM. ✦ *p* < 0.05 IMQ mice or IMQ-POE mice vs. control mice by one-way ANOVA with Bonferroni post hoc test; ✶ *p* < 0.05 IMQ-POE mice vs. IMQ mice by one-way ANOVA with Bonferroni post hoc test.

To check whether the characteristic features of psoriasis were also present at the microscopic level, at the end of the five days of treatment, we performed a histological examination by hematoxylin and eosin staining of the dorsal skin area treated with vehicle cream (control mice), or with IMQ (IMQ mice), or with IMQ and oral administration of POE (IMQ-POE mice). In comparison to control mice (Figure 5a), IMQ topical application induced in both IMQ and IMQ-POE mice the characteristic psoriatic changes of the stratified squamous epithelium [32] represented by (a) acanthosis (Figure 5b,c, black arrowheads), i.e., epidermal hyperplasia with an increased thickness of stratum spinosum; (b) hyperkeratosis and parakeratosis (Figure 5b,c, double black arrowheads), i.e., thickening of the stratum corneum and retention of nuclei in the stratum corneum; (c) agranulosis (Figure 5b,c, white arrowheads), i.e., reduction in the stratum granulosum. However, when epidermal hyperplasia was evaluated by measurement of epidermal thickness, excluding stratum corneum and rete pegs, during oral administration of POE, there was a statistically significant reduction (*p* < 0.05) in epidermal hyperplasia in IMQ-POE mice compared to that observed in IMQ mice (Figure 5d, 59.4 ± 1.5 μm versus 90.1 ± 2.7 μm, *p* < 0.05), although oral administration of POE was not able to completely revert normal epidermal thickness observed in control mice (Figure 5d, 23.9 ± 0.7 μm). In addition, during oral administration of POE, the height of dermal papillae was reduced in IMQ-POE mice compared to IMQ mice (Figure 5e, 86.0 ± 2.8 μm versus 120.0 ± 1.9 μm, *p* < 0.05), although it remains greater than control mice (Figure 5e, 25.1 ± 0.6 μm). Furthermore, while the signs of skin inflammation were evident in IMQ mice, they were largely absent in specimens of lesional skin of IMQ-POE mice. Indeed, the lesional skin of IMQ mice showed dilatation and congestion of the dermal capillaries (Figure 5b, black arrows), and the presence of Munro’s microabscesses, i.e., aggregates of pyknotic neutrophils in the stratum corneum (Figure 5b, double white arrowheads), which are one of the hallmarks of early psoriasis [32]. The improvement of psoriatic inflammatory characteristics induced by oral administration of POE was also confirmed by ELISA detection of the CD86 marker of inflammatory macrophages and CD3 marker of lymphocytes in the skin specimens. As shown in Figure 5e, while in control mice, CD86 and CD3 markers were almost undetectable, in IMQ mice, the levels of both CD86 and CD3 markers were expressed at a higher level than in IMQ-POE mice (CD86, 0.9 ± 0.01 pg/mg versus 0.7 ± 0.008 pg/mg, *p* < 0.05; CD3 2.2 ± 0.07 pg/mg versus 1.8 ± 0.02 pg/mg, *p* < 0.05). 

### 2.4. Evaluation of Cytokines and LCN-2 Expression

To verify the effect of oral administration of POE on the expression of IL-23 and TNF-α, of the Th17-associated IL-17A and IL-17F cytokines, of Th1-associated IFN-γ and IL-2 cytokines, the protein expression of cytokines was evaluated by ELISA in the treated dorsal skin area and in plasma at the end of the experimental period (day 5).

**Figure 6:**
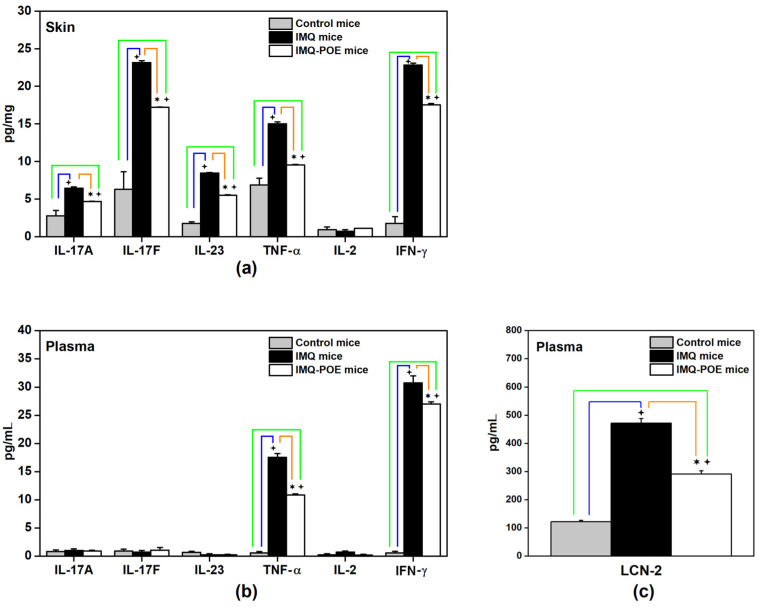
Protein expression of cytokines and LCN-2. The levels of cytokines and LCN-2 were measured by ELISA in control mice (grey bar), IMQ mice (black bar), and IMQ-POE mice (white bar). (**a**) The levels of IL-17A, IL-17F, IL-23, TNF-α, IL-2, and IFN-γ in the treated dorsal skin area are reported in pg/mg of tissue; (**b**) the level of IL-17A, IL-17F, IL-23, TNF-α, IL-2, and IFN-γ and (**c**) of LCN-2 in plasma is reported in pg/mL. Values are expressed as mean ± SEM (*n* = 6/group). ✦ *p* < 0.05 IMQ mice or IMQ-POE mice vs. control mice by one-way ANOVA with Bonferroni post hoc test; ✶ *p* < 0.05 IMQ-POE mice vs. IMQ mice by one-way ANOVA with Bonferroni post hoc test.

As shown in Figure 6a, protein expression of IL-23 and TNF-α, of the Th17-associated cytokines IL-17A and IL-17F, and of the Th1-associated IFN-γ was higher in the dorsal treated skin area of IMQ and IMQ-POE mice than in control mice, while only the Th1-associated IL-2 was almost unexpressed in skin samples. However, all these cytokines were lower in IMQ-POE mice than in IMQ mice (IL-23, 5.5 ± 0.06 pg/mg versus 8.4 ± 0.08 pg/mg, *p* < 0.05; TNF-α, 9.5 ± 0.08 pg/mg versus 14.9 ± 0.2 pg/mg, *p* < 0.05; IL-17A, 4.6 ± 0.04 pg/mg versus 6.4 ± 0.2 pg/mg, *p* < 0.05; IL-17F, 17.1 ± 0.04 pg/mg versus 23.1 ± 0.2 pg/mg, *p* < 0.05; IFN-γ, 17.5 ± 0.17 pg/mg versus 22.8 ±0.2 pg/mg, *p* < 0.05).

In plasma (Figure 6b), the expression of IL-23, IL-17A, IL-17F, and IL-2 was almost undetectable in all three groups of mice, and only TNF-α and IFN-γ were more highly expressed in IMQ mice and in IMQ-POE mice than in control mice. However, in IMQ mice, TNF-α and IFN-γ were expressed at a higher level than in IMQ-POE mice (TNF-α 17.5 ± 0.6 pg/mL versus 10.8 ± 0.2 pg/mL, *p* < 0.05; IFN-γ, 30.7 ± 1.2 pg/mL versus 27 ± 0.3 pg/mL, *p* < 0.05).

Detection of LCN-2 expression in plasma samples by ELISA at the end of the experimental period revealed that LCN-2 protein expression was higher in IMQ mice and in IMQ-POE mice compared to control mice. Moreover, in IMQ mice, LCN-2 concentration was about twice that in IMQ-POE mice (Figure 6c, 472.1 ± 15.6 pg/mL versus 291.6 ± 11.1 pg/mL, *p* < 0.05). These results further support the role of POE as a mitigator of proinflammatory effects associated with psoriatic lesions.

## 3. Discussion

Psoriasis is an immune-mediated chronic inflammatory dermatological and systemic disease influenced by genetic predisposition and environmental stimuli. The main histological characteristic is a dysregulated proliferation and differentiation of keratinocytes and infiltration of inflammatory cells such as T lymphocytes, neutrophils, and macrophages. Interactions between keratinocytes and immune cells play a pivotal role in the pathogenesis of psoriatic lesions. Keratinocytes activated by environmental and microbial stimuli secrete cytokines and antimicrobial peptides that activate dendritic cells, which in turn activate subsets of Th lymphocytes that release several cytokines such as IL-23, IL-17A, and IL-17F and TNF-α, which can promote proliferation and altered differentiation of keratinocytes, creating a loop of maintenance and amplification of inflammation [33,34]. Therefore, for the treatment of psoriasis, especially for the more serious forms, in recent decades, there has been a great development of biological drugs based on the use of antibodies against cytokines, particularly against TNF-α, IL-17A, IL-17F, and IL-23, which are not only involved in skin lesions, but they can also have systemic effects [18,35,36,37]. However, the use of biological drugs can be limited by several factors, such as the risk of immunogenicity [20,38,39], long-term administration, which can affect patient compliance, and the expensive cost. Thus, researchers’ attention has recently been focused on the anti-inflammatory and immune response modulation activities of various natural products with the aim of helping traditional therapies improve patient’s safety and adherence to treatment [40,41,42].

Modern science has recognized the millennia-long use of plants by humans to fight disease. *Posidonia oceanica* is a marine plant used since the time of ancient Egypt to treat various human ailments and for skin care. Our research group has studied the bioactive properties of a hydroalcoholic extract of *P. oceanica* leaves (POE) for years. Although the phytochemical extracts are highly heterogeneous, due to the presence of a mixture of different bioactive components, it has been observed that biological responses may not be due to a single bioactive compound, but to the mix of several bioactive compounds [43,44,45,46]. Furthermore, recent studies have revealed that proanthocyanidins, derived from grapes, apples, and other vegetables, are composed of a group of natural polyphenols that can alleviate psoriatic inflammation [47,48], supporting the role of synergism between bioactive compounds. The analysis of POE by UPLC allowed us to reveal that POE is made up of 88% polyphenols and is predominantly represented by (+) catechins and to a lesser extent by gallic acid, ferulic acid, epicatechin, and chlorogenic acid [49]. Nevertheless, we have revealed that neither single compounds nor even catechin alone can explain the biological effects of the entire *Posidonia oceanica* extract. [49]. Both in vitro and in vivo scientific studies of the leaf extract of this marine plant have shown that the phytocomplex can be exploited in various human health applications due to its nontoxicity [24]. Among other bioactivities, the anti-inflammatory role of POE has been described both in vitro and in vivo [27,28]. Because inflammation plays an important role in psoriasis, in this preliminary study, we have investigated the beneficial role of POE in the pathogenesis of psoriatic-like lesions in an IMQ-induced mouse model of psoriasis. We have chosen the oral administration route of POE, both due to the absence of its toxicity in vivo [28] and because psoriasis is now considered a systemic disease.

In this study, we have provided evidence that oral administration of POE can inhibit psoriasiform dermatitis induced in mice by IMQ treatment, an approved animal model for the in vivo experimental study of psoriasis [14,15]. 

First, PASI score, skin thickness, and lesional skin temperature, a cardinal sign of inflammation, were reduced in mice treated by oral administration of POE, indicating that POE can effectively reduce the classical clinical aspects of psoriatic skin lesions. Moreover, POE was able to inhibit the hallmark histological aspects of psoriasiform dermatitis, such as thickness of stratified squamous epithelium, hyperkeratosis, parakeratosis, agranulosis, dilatation, and congestion of the dermal capillaries and development of Munro’s micro abscesses [32], and to reduce infiltration of inflammatory macrophages and T lymphocytes, as revealed by reduction in expression of CD86 and CD3 markers. These inhibitory effects are consistent with previous results from our laboratory which revealed that POE can reduce signs and symptoms of inflammation such as inflammatory pain and oedema in CD1 mice [28]. Furthermore, oral administration of POE in IMQ-treated mice was able to reduce the clinical and histopathological signs of psoriasis without additional systemic adverse effects, as demonstrated by the absence of significant changes in the relative organ weights of the liver and kidney compared to the control mice.

Second, POE was also able to inhibit, in lesional skin of IMQ mice, protein expression of the major inflammatory cytokines implicated in the pathogenesis of psoriasis, such as the Th1 hallmark cytokine TNF-α and IFN-γ and the Th17 product IL-17A and IL-17F, and the cytokine IL-23 secreted by dendritic cells, which activates Th1 and Th17 cells to produce IL-17, IFN-γ, and TNF-α. In addition, POE was able to reduce plasma levels of inflammatory cytokines TNF-α and IFN-γ, whose high serum levels correlate with disease severity in psoriatic patients [50]. These results agree with our previous study that revealed the inhibitory effect of POE on the protein expression of cytokines TNF-α and IL-1 in another murine inflammatory model [28]. Several biological effects of TNF are mediated by NF-κB activation [51], including cellular proliferation, differentiation, and apoptosis, so that NF-κB is considered an essential nuclear transcription factor in the pathogenesis of psoriasis [52]. Recently, our laboratory has shown that POE is able to inhibit NF-κB activation by preventing phosphorylation and nuclear translocation of the p65 subunit of NF-κB through inhibition of IκBα degradation [27]. 

In this regard, it might be interesting to extend our study on the role of POE in the immune-mediated pathogenesis of psoriasis not only by inhibiting upstream signaling agents, such as TNF expression, but also by inhibiting downstream signaling agents of the canonical NF-κB pathway. Therefore, we plan to verify in a future study the role of POE in an in vitro model of psoriasis using both the hyperproliferative model of LPS/IL-22-stimulated HaCaT cells and the inflammatory model of HaCaT cells stimulated with LPS/TNF-α [53,54].

Finally, in our psoriasis mouse model, we have revealed that plasma levels of LCN-2 were lowered during oral administration of POE. LCN-2 has recently been proposed as a potential target for psoriasis treatment, since it is involved in keratinocyte differentiation and pathogenesis of psoriasis by stimulating neutrophil function, Th17 activation, and secretion of IL-23 by dendritic cells [11]. Psoriatic patients display a higher risk of developing nonmelanoma skin cancer than healthy subjects, especially those patients with moderate to severe psoriasis [55]. Recently, it has been revealed that plasma levels of LCN-2 were significantly higher in psoriatic patients with nonmelanoma skin cancer than in patients with skin tumours without psoriasis [56]. Therefore, POE might also be involved in reducing the risk of developing skin cancers in psoriatic patients by inhibiting LCN-2 expression.

In conclusion, our preliminary study proposes POE as a promising natural source of bioactive compounds that might improve traditional pharmacological treatment of psoriasis.

## 4. Materials and Methods

### 4.1. Chemicals and Reagents

All chemicals and reagents were purchased from Sigma-Aldrich (Merck KGaA, Darmstadt, Germany) unless otherwise stated. 

### 4.2. Animals and Ethical Statement

C57BL/6 mice were purchased from Envigo (Varese, Italy). Experiments were conducted with female C57BL/6 mice 10 weeks old, weighing 25–35 g at the beginning of the experimental procedure. Animals were housed in conventional cages in the CeSAL animal facility (Centro Stabulazione Animali da Laboratorio, University of Florence) under controlled environmental conditions (room temperature at 22 °C and 12 h light/12 h dark cycle) and provided with commercial solid food and tap water available ad libitum. All animal manipulations were carried out according to Directive 2010/63/EU of the European Parliament and of the European Union Council (22 September 2010) on the protection of animals used for scientific purposes. The ethical policy of the University of Florence complies with the Guide for the Care and Use of Laboratory Animals of the US National Institutes of Health (NIH Publication No. 85–23, revised 1996; University of Florence assurance number: A5278-01). All the experimental procedures carried out in this study were approved by the Italian Ministry of Health (No. 333/2020-PR) and by the Animal Subjects Review Board of the University of Florence. Experiments involving animals were performed according to the ARRIVE guidelines [57,58]. All efforts were made to minimize animal suffering and to reduce the number of animals used.

### 4.3. Preparation of Posidonia oceanica Leaf Extract (POE)

*P. oceanica* leaves were collected and extracted as previously described [49]. Briefly, 1 g of *P. oceanica* dried leaves after mincing was suspended in 10 mL EtOH/H2O (70:30 *v*/*v*) at 37 °C under stirring overnight and then stirred at 65 °C for an additional 3 h. After repeated centrifugations, the hydroalcoholic phase was mixed in a 1:1 ratio with n-hexane (*v*/*v*) under shaking. Then, the recovered hydroalcoholic fraction was dispensed in 1 mL aliquots and dried. The dry extract will be called POE. Specifically, in this study, the hydroalcoholic extract from 4 g of *P. oceanica* dried and minced leaves gave a yield of 0.2 g of POE. In order to analyze the biochemical composition of POE, 9.4 mg of dry extract was solubilized in 0.5 mL of EtOH/H2O (70:30 *v*/*v*) and POE was then assessed for total polyphenol content (Folin–Ciocâlteu method), and for its antioxidant (FRAP assay) and free-radical scavenging (DPPH assay) activities, as previously described [59]. The total polyphenol content in POE was 0.5 ± 0.06 mg/mL of gallic acid equivalents, and the antioxidant and radical-scavenging activities were 0.13 ± 0.07 mg/mL and 1.1 ± 0.2 mg/mL of ascorbic acid equivalents, respectively.

### 4.4. Imiquimod-Induced Psoriasis and POE Treatment

**Scheme 1:**
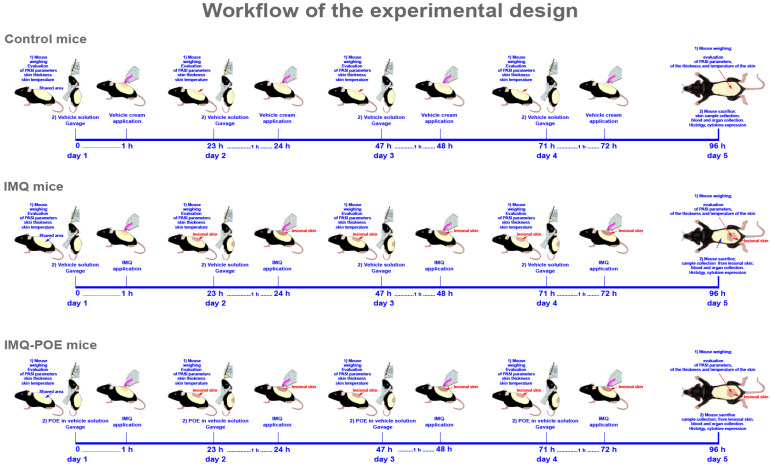
Illustration of the experimental design.

The animals were divided into 2 groups: (a) the control mice (*n* = 6) and (b) the experimental mice (*n* = 12). All the mice were housed individually, with 1 animal per cage to prevent licking each other. Psoriasis-like lesions were induced in the experimental mice according to the protocol employed in previous studies [14,15,60]. Briefly, a dose of 62.5 mg of commercially available Imiquimod 5% cream (Imunocare; Difa Cooper S.p.A., Milan, Italy), corresponding to 3.125 mg of Imiquimod, was topically applied daily on a 2 cm^2^ shaved dorsal skin area for 5 consecutive days. This dose of IMQ was already demonstrated in various publications [14,15,60] to induce reproducible psoriatic-like skin inflammation in this mouse model. To analyze the effect of POE during treatment with IMQ, the experimental mice were divided into two subgroups: (a) the group of so-called IMQ mice (*n* = 6) treated topically with IMQ, which were orally administered by gastric gavage with vehicle solution (1% carboxymethyl cellulose sodium salt, CMC) at a volume of 10 mL/kg of the animal weight 1 h before each topical treatment with IMQ, and (b) the group of so-called IMQ-POE mice (*n* = 6), to which, 1 h before each topical treatment with IMQ, POE resuspended in the vehicle solution was orally administered by gastric gavage at a dose of 100 mg/kg of the animal body weight. The oral POE dosage at 100 mg/kg was chosen based on the anti-inflammatory effects of POE observed in mice in our previous study [28]. The control group was treated similarly by topical application of the vehicle cream (isostearic acid, benzyl alcohol, cetyl alcohol, stearyl alcohol, soft white paraffin, polysorbate 60, sorbitan stearate, glycerol, methyl hydroxybenzoate (E218), propyl hydroxybenzoate (E216), xanthan gum, purified water), and by oral administration with vehicle solution 1 h before each topical treatment with vehicle cream. The body weight of each group of mice was recorded every day starting on the day of application of each cream (day 1) until the end of the experimental period (day 5). To facilitate comprehension of the experimental design, we have provided a schematic workflow in Scheme 1.

### 4.5. Assessment of Inflammation Severity

In order to compare the severity of psoriatic-like lesions between control mice, IMQ mice, and IMQ-POE mice, a modified PASI score was assessed every day starting on day 1 until the end of the experiment (day 5). This evaluation was performed before the above daily treatments in all three groups of mice. Except for the extension of the area affected by psoriatic-like lesions, since the extension of the experimental treated area was the same and it was not related to spontaneously occurring psoriasis, a scale from 0 to 4 was used to evaluate erythema, desquamation, and infiltration, where 0 = none, 1 = mild, 2 = moderate, 3 = severe, and 4 = very severe. The cumulative index (sum of the three scores) was then evaluated as a measure of the overall inflammation severity comparable to the PASI score (maximum score = 12). In addition, we measured the double-fold dorsal skin thickness of the treated area using an electronic digital caliper, resolution: 0.01mm (150 mm Stainless Steel Electronic Digital Calliper, Juning, Shenzhen, Guangdong 518000, China). Finally, the temperature of the dorsal treated skin area was measured daily by an infrared camera (Pti120, Fluke s.r.l., Milan, Italy) during the 5 days of treatment in all three groups of mice. Images were analyzed by Fluke Connect Desktop Software (version 4.3)

### 4.6. Organ and Blood Collection and Tissue Histological Examination

At the end of the 5th day of treatment, mice were anesthetised with isoflurane (2%), and the blood was collected from the vena cava in the presence of 2% EDTA as anticoagulant and centrifuged (2500 RCF for 10 min at room temperature) to obtain the plasma fraction; mice were then sacrificed by decapitation. The spleen, liver, and kidneys were immediately removed and weighed. The organ relative weight was calculated by the ratio of each animal’s organ weight to body weight and expressed as the percentage of body weight. In addition, biopsies were taken from vehicle cream-treated skin areas in control mice and from Imiquimod-treated skin areas in IMQ and IMQ-POE mice by surgical excision from each mouse. Briefly, skin specimens were fixed in 10% phosphate-buffered formalin, dehydrated, cleared, and then embedded in paraffin. Skin sections (5 μm) were cut, mounted on glass slides, deparaffinized, and stained with hematoxylin and eosin. The mounted specimens were then observed under a bright-field light microscope (BX40 upright microscope, Olympus, Tokyo, Japan) equipped with a digital microscope camera (C B5, Optika, Bergamo, Italy). Epidermal thickness, excluding stratum corneum and rete pegs, and the height of dermal papillae, measured by the distance between the top of dermal papillae and the level showing no dermal papillae, were determined in 20 randomly chosen microscopic fields in each section from each mouse specimen using Aperio ImageScope software version 12.4.6 (Leica Biosystems, Nußloch, Germany).

### 4.7. Quantitation of Cytokines and LCN-2 Protein Expression

Levels of TNF-α, and IL-23, and of Th17-associated cytokines IL-17A and IL-17F, and of Th1-associated cytokines IFN-γ and IL-2 were measured by enzyme-linked immunosorbent assay (ELISA) in skin tissue samples and in plasma. 

Proteins were extracted from skin sections at the end of the experimental period according to the method described by Kawashima et al. [61]. Briefly, twenty serial sections from each paraffin-embedded specimen were placed in 1.5 mL vials and deparaffinized by incubation for 10 min at room temperature in xylene and centrifuged for 3 min at 16,000× *g*. The pellet was resuspended in xylene for an additional 10 min incubation and centrifuged again. The pellet was then hydrated in a series of graded ethanol, air-dried, and resuspended in 50 μL of lysis buffer (400 mM Tris–HCl pH 8.0, 150 mM NaCl, 1 mM EGTA, 1 mM EDTA, 1% Triton X-100, 0.5% sodium deoxycholate, 50 mM NaF, 1 mM PMSF, 1 mM AEBSF, 800 nM Aprotinin, 50 μM Bestatin, 15 μM E64, 20 μM Leupeptin, 10 μM Pepstatin A), homogenized using a micro pestle (Carl Roth GmbH + Co. KG), and incubated at 90 °C for 60 min. The debris was removed by centrifugation, and the total protein concentration in tissue lysates was determined by Lowry’s method [62] using bovine serum albumin as a reference.

The expression of TNF-α, IL-23, IL-17A, IL-17F, IFN-γ, and IL-2 in tissue supernatant and in plasma was measured using commercial ELISA assay kits (TNF alpha Mouse Uncoated ELISA Kit, IL-12/IL-23 total p40 Uncoated ELISA kit, IL-17A homodimer Mouse Uncoated ELISA kit, IL-17F homodimer Mouse Uncoated ELISA kit, IFN-γ Mouse Uncoated ELISA kit, IL-2 Mouse Uncoated ELISA kit; Invitrogen Thermo Fisher Scientific, Inc., Waltham, MA, USA) following the standard protocol provided by the assay kit manufacturer. Concentrations were expressed as picogram of cytokine per milligram of extracted protein from tissue samples or as picogram of cytokine per milliliter of plasma.

Lipocalin-2 concentration was examined in plasma at the end of the experimental period by a commercial ELISA kit (Mouse NGAL ELISA Kit, Bioporto Diagnostics A/S, Hellerup, Denmark) according to manufacturer instructions. Concentrations were expressed as picogram of LCN-2 per milliliter of plasma.

### 4.8. Immunodetection of T-Lymphocytes and Macrophages in Skin Sections 

Macrophage and lymphocyte T infiltration in vehicle cream-treated skin areas of control mice and in Imiquimod-treated skin areas of IMQ and IMQ-POE mice was evaluated by detecting the CD86 marker for inflammatory macrophages and the CD3 marker for T cells. Briefly, the expression of CD86 and CD3 was measured in tissue supernatant isolated from the paraffin-embedded specimen, as described above using commercial ELISA assay kits (CD86 ELISA Kit, CD3 ELISA Kit; Antibodies-online, GmbH, Aachen, Germany) according to manufacturer instructions. Concentrations were expressed as nanogram per milligram of extracted protein.

### 4.9. Statistical Analysis

Results were expressed as the mean ± SEM and were analyzed statistically using JASP software (JASP Team 2024 Version 0.18.3). The statistical difference between groups was analyzed using one-way ANOVA with a Bonferroni correction (Bonferroni post hoc test); statistical significance was defined as *p* < 0.05.

## Data Availability

The data presented in this study are available on request from the corresponding author.
